# Moderate Physical Activity Increases the Expression of ADNP in Rat Brain

**DOI:** 10.3390/ijms25084382

**Published:** 2024-04-16

**Authors:** Grazia Maugeri, Agata Grazia D’Amico, Concetta Federico, Salvatore Saccone, Velia D’Agata, Giuseppe Musumeci

**Affiliations:** 1 Section of Anatomy, Histology and Movement Sciences, Department of Biomedical and Biotechnological Sciences, University of Catania, 95100 Catania, Italy; graziamaugeri@unict.it (G.M.); g.musumeci@unict.it (G.M.); 2 Department of Drug Sciences, University of Catania, 95125 Catania, Italy; agata.damico@unict.it; 3 Section of Animal Biology, Department of Biological, Geological and Environmental Sciences, University of Catania, 95123 Catania, Italy; federico@unict.it (C.F.); saccosal@unict.it (S.S.)

**Keywords:** ADNP, β-Tubulin III, moderate physical activity, brain, dentate gyrus, cerebellum

## Abstract

Activity-dependent neuroprotective protein (ADNP) is a neuroprotective protein essential for embryonic development, proper brain development, and neuronal plasticity. Its mutation causes the autism-like ADNP syndrome (also called the Helsmoortel-Van der Aa syndrome), characterized by neural developmental disorders and motor dysfunctions. Similar to the ADNP syndrome, the *ADNP* haploinsufficient mouse shows low synapse density, leading to motor and cognitive ability delays. Moderate physical activity (PA) has several neuroprotective and cognitive benefits, promoting neuronal survival, differentiation, neurogenesis, and plasticity. Until now, no study has investigated the effect of moderate exercise on ADNP expression and distribution in the rat brain. The aim of the current investigation was to study the effects of moderate exercise on the ADNP expression and neuronal activation measured by the microtubule protein β-Tubulin III. In pursuit of this objective, twenty-four rats were selected and evenly distributed into two categories: sedentary control rats and rats exposed to moderate physical activity on a treadmill over a span of 12 weeks. Our results showed that moderate PA increases the expression of ADNP and β-Tubulin III in the dentate gyrus (DG) hippocampal region and cerebellum. Moreover, we found a co-localization of ADNP and β-Tubulin III in both DG and cerebellum, suggesting a direct association of ADNP with adult neuronal activation induced by moderate PA.

## 1. Introduction

Moderate physical activity (PA) is an indispensable element in promoting the all-round well-being of an individual. It is known to elicit several beneficial effects at the systemic level, by preventing cardiovascular diseases, inflammation, osteoporosis, and muscle atrophy [1]. Regular exercise positively affects psychological well-being and glucose metabolism, by reducing the developing of type 2 diabetes in high-risk individuals. Furthermore, the effects of moderate PA are also relevant in mental disorders, cancers, and neurodegenerative diseases [2,3,4,5]. There is also growing recognition that moderate PA plays a key role in “brain rejuvenation”. At molecular and cellular levels, PA promotes the expression of neurotransmitter and neurotrophic factors associated with neurogenesis [6,7,8]. Moreover, the positive role on cognitive functions such as learning, memory, and executive functions is also due to the direct association between PA and hippocampal volume [9,10]. In particular, several studies have demonstrated that moderate PA promotes neural plasticity in the dentate gyrus (DG), by increasing DG cerebral blood volume [11,12,13,14]. Moderate exercise also promotes cerebellar motor functions, by improving mitochondrial functions and counteracting the loss of Purkinje cells [15,16,17].

Activity-dependent protein (ADNP) is a neuroprotective protein whose gene was discovered in 1999 by Bassan et al. [18]. The peptide was found in nervous systems and different organs [19,20]. In fact, ADNP is indispensable for brain formation [21] and, through the interaction with nuclear chromatin, regulates the transcription of several genes linked to autophagy, axonal transport, embryogenesis, dendritic spine plasticity, and autism-linked protein translation [22,23,24]. Somatic mutations in the *ADNP* gene are detected in the brains of individuals affected by Alzheimer’s disease. Furthermore, de novo mutations in the *ADNP* gene are associated with ADNP syndrome (also known as Helsmoortel Van Der Aa syndrome), which manifests a variety of clinical symptoms, including global developmental delays, autistic/intellectual disability, and motor dysfunctions [25,26,27,28]. ADNP immunoreactivity has been detected in various brain regions, including the hippocampus and cerebellum, suggesting the key role of ADNP in brain functioning [29].

In 1999, NAP (NAPVSIPQ), a short peptide containing eight amino acids of ADNP and mimicking its functions, was synthesized [18]. The exogenous administration of NAP has been shown to exert a beneficial role in different pathological conditions, such as severe head injury, cerebral ischemia, and ocular damage [28,29,30,31,32,33,34]. In the brain, NAP treatment induces neuroplasticity and neuroprotection through microtubule (MT) reorganization. Its treatment promotes the MT network area/cell and increases the expression of β-Tubulin III. The latter is a neuronal marker in the developing and mature human nervous system [30]. β-Tubulin III regulates early neuritogenesis in association with other microtubule (MT)-associated proteins (MAPs). Moreover, it is involved in neurite extension by inducing MT polymerization in early neuritogenesis [31].

Given the significant neuroprotective role of moderate PA in promoting neuronal survival, neurogenesis, and plasticity, we investigated its impact on ADNP expression, considering that this protein exerts a key role in brain formation and maintenance [21]. We analyzed its expression in the dentate gyrus (DG) of the hippocampus and cerebellum, since (1) both areas express ADNP in basal conditions [29] and (2) physical exercise exerts profound effects on the hippocampal and cerebellar structures [32,33]. Here, we report for the first time that ADNP was significantly up-regulated in the DG and cerebellum of rats performing moderate PA. Additionally, extensive co-expression was discovered for ADNP and the marker for neuronal differentiation β-tubulin III. Taken together, the results suggest that the protective role of moderate PA could be partly mediated by up-regulation and interaction between ADNP and β-Tubulin III in specific brain areas.

## 2. Results

### 2.1. Moderate PA Increases β-Tubulin III Expression in Both the Rat Dentate Gyrus and Cerebellum

**Figure 1:**
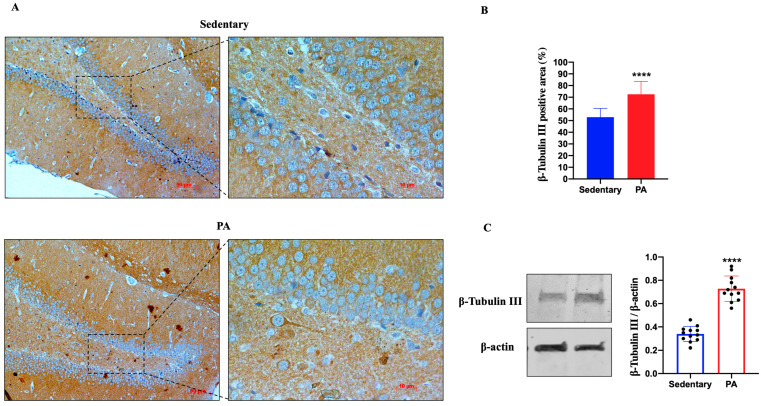
β-Tubulin III expression in DG of group 1 (sedentary) and group 2 (PA). (**A**). Immunodetection of β-Tubulin III in DG of sedentary (N = 12) and PA (N = 12) rats. The digital micrographs provided are representative outcomes of fields captured from randomly selected slides. These images were acquired utilizing the Zeiss Axioplan light microscope (Carl Zeiss, Oberkochen, Germany) equipped with a digital camera (AxioCam MRc5; Carl Zeiss). Scale bar: 50 μm and 10 μm. (**B**). The bar graph shows the percentage of β-Tubulin III positive area after IHC detection conducted across three separate experiments. Data represent means ± SEM. **** *p* < 0.0001 vs. sedentary, as determined by unpaired two-tailed Student *t*-test. (**C**). Representative immunoblots of β-Tubulin III expression in hippocampus of sedentary (N = 12) and PA (N = 12) rats. The bar graph illustrates the quantitative assessment of signals derived from immunoblots conducted across three separate experiments. Relative band densities were measured using ImageJ software. Protein levels are presented in arbitrary units after normalization to β-actin, serving as the loading control. Data represent means ± SEM. **** *p* < 0.0001 vs. sedentary, as determined by unpaired two-tailed Student *t*-test.

Figure 1A shows the representative IHC results showing β-Tubulin III expression in the dentate gyrus (DG) of sedentary and active rats. Moderate β-Tubulin III staining was detected in the DG of sedentary rats, whereas higher expression was found in the DG of active rats, suggesting that moderate PA can positively influence the expression of β-Tubulin III. Therefore, we also investigated the expression of β-Tubulin III in the serial brain sections (coming from the same paraffin-embedded samples used for IHC analysis) of sedentary and moderate PA rats through Western blot analysis. As shown in Figure 1B, analogous to the results of IHC, β-Tubulin III expression levels were significantly increased in the DG of rats performing moderate PA.

**Figure 2:**
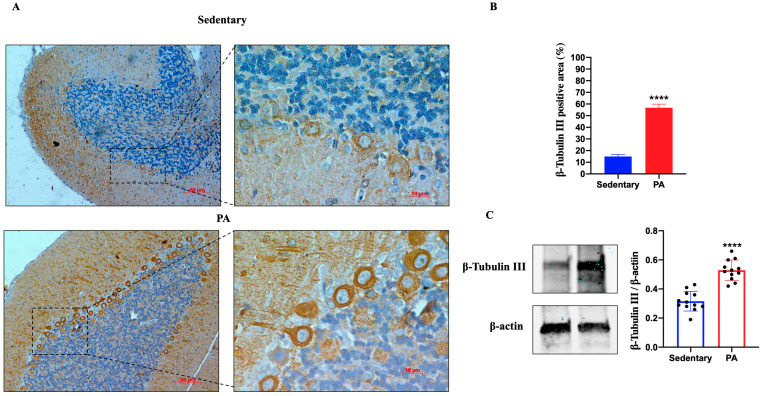
β-Tubulin III expression in cerebellum of group 1 (sedentary) and group 2 (PA). (**A**). Immunodetection of β-Tubulin III in cerebellum of sedentary (N = 12) and PA (N = 12) rats. Digital micrographs are representative results of fields taken in randomly selected slides and obtained using the Zeiss Axioplan light microscope, fitted with a digital camera. Scale bar: 50 μm and 10 μm. (**B**). The bar graph shows the percentage of β-Tubulin III positive area after IHC detection resulting from three independent experiments. Data represent means ± SEM. **** *p* < 0.0001 vs. sedentary, as determined by unpaired two-tailed Student *t*-test. (**C**). Representative immunoblots of β-Tubulin III expression in cerebellum of sedentary (N = 12) and PA (N = 12) rats. The bar graph shows quantitative analysis of signals obtained by immunoblots resulting from three independent experiments. Relative band densities were quantified using ImageJ software. Protein levels are expressed as arbitrary units obtained following normalization to β-actin, which was used as loading control. Data represent means ± SEM. **** *p* < 0.0001 vs. sedentary, as determined by unpaired two-tailed Student *t*-test.

We further investigated the effect of moderate PA on β-Tubulin III expression in the cerebellum of sedentary and active rats. As shown in Figure 2A, moderate β-Tubulin III staining was detected in the molecular layer, Purkinje cell layer, and granular layer of the sedentary rats. Similar to the hippocampus data, the brightest β-Tubulin III was found in the cerebellum of rats performing moderate PA, particularly in the dendrites and cell bodies of Purkinje cells. The IHC results were also confirmed by Western blot analysis. In fact, β-Tubulin III protein expression was significantly increased in moderate PA rats as compared to sedentary rats.

### 2.2. Moderate PA Increases ADNP Expression in Both the Rat DG and Cerebellum

**Figure 3:**
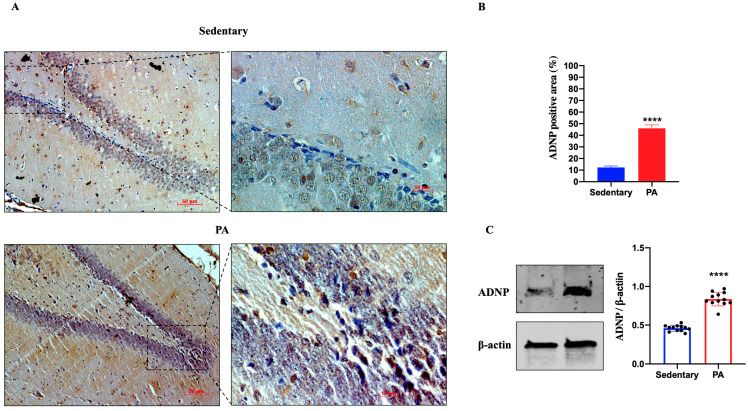
ADNP expression in DG of group 1 group 1 (sedentary) and group 2 (PA). (**A**). Immunodetection of ADNP in DG of sedentary (N = 12) and PA (N = 12) rats. Digital micrographs are representative results of fields taken in randomly selected slides and obtained using the Zeiss Axioplan light microscope, fitted with a digital camera. Scale bar: 50 μm and 10 μm. (**B**). The bar graph shows the percentage of ADNP positive area after IHC detection resulting from three independent experiments. Data represent means ± SEM. **** *p* < 0.0001 vs. sedentary, as determined by unpaired two-tailed Student *t*-test. (**C**). Representative immunoblots of ADNP expression in hippocampus of sedentary (N = 12) and PA (N = 12) rats. The bar graph shows quantitative analysis of signals obtained by immunoblots resulting from three independent experiments. Relative band densities were quantified using ImageJ software. Protein levels are expressed as arbitrary units obtained following normalization to β-actin, which was used as loading control. Data represent means ± SEM. **** *p* < 0.0001 vs. sedentary, as determined by unpaired two-tailed Student *t*-test.

Next, we investigated the effect of moderate PA on the expression of ADNP in the rat DG and cerebellar cortex. As shown in Figure 3A, ADNP immunostaining was detected in the DG of sedentary rats. However, higher ADNP immunoreactivity was found in the DG of active rats. These data were confirmed by Western blot analysis (Figure 3B), showing a significant ADNP expression in the DG of moderate PA rats as compared to sedentary ones.

**Figure 4:**
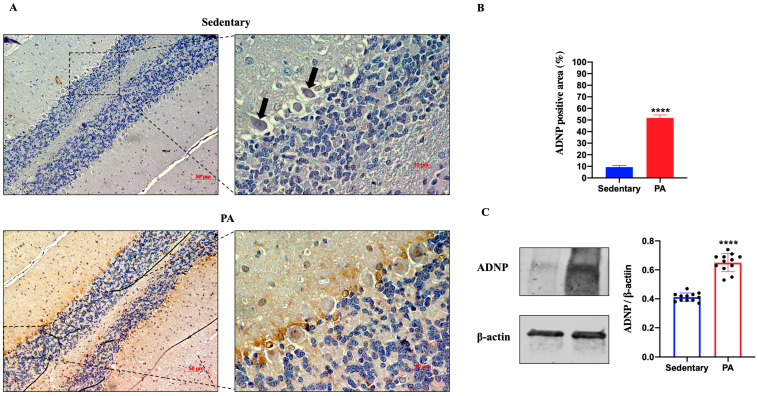
ADNP expression in cerebellum of group 1 (sedentary) and group 2 (PA). (**A**). Immunodetection of ADNP in cerebellum of sedentary (N = 12) and PA (N = 12) rats. Digital micrographs are representative results of fields taken in randomly selected slides and obtained using the Zeiss Axioplan light microscope, fitted with a digital camera. Scale bar: 50 μm and 10 μm. The black arrows indicate the spot of ADNP immunoreactivity (**B**). The bar graph shows the percentage of ADNP positive area after IHC detection resulting from three independent experiments. Data represent means ± SEM. **** *p* < 0.0001 vs. sedentary, as determined by unpaired two-tailed Student *t*-test. (**C**). Representative immunoblots of ADNP expression in cerebellum of sedentary (N = 12) and PA (N = 12) rats. The bar graph shows quantitative analysis of signals obtained by immunoblots resulting from three independent experiments. Relative band densities were quantified using ImageJ software. Protein levels are expressed as arbitrary units obtained following normalization to β-actin, which was used as loading control. Data represent means ± SEM. **** *p* < 0.0001 vs. sedentary, as determined by unpaired two-tailed Student *t*-test.

Then, we investigated the effect of moderate PA on ADNP expression in the rats’ cerebellum. As shown in Figure 4A, faint ADNP immunoreactivity was detected in the cerebellum of sedentary rats, limited to the nucleus of Purkinje cells (black arrows). On the other hand, higher ADNP staining was detected in the cerebellar cortex of active rats, particularly in the Purkinje cell layer. These IHC results were confirmed by Western blot analysis. Indeed, as shown in Figure 4B, the ADNP expression was significantly increased in the cerebellum of rats performing moderate PA as compared to sedentary animals.

### 2.3. Moderate PA Induces Co-Localization of ADNP and β-Tubulin III in Rat DG and Cerebellum

**Figure 5:**
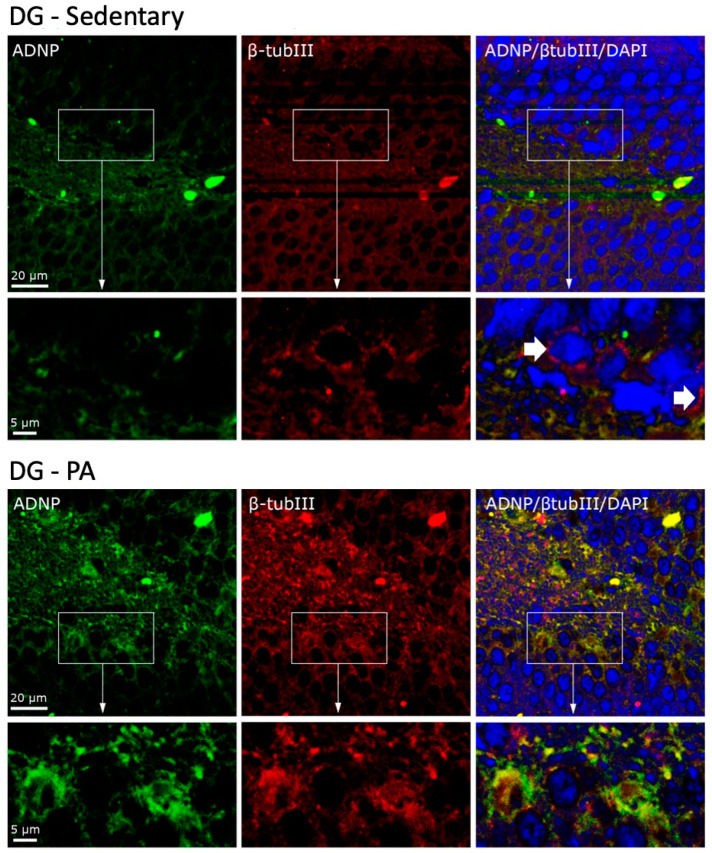
Co-localization of β-Tubulin III and ADNP in DG of sedentary (N = 12) and active rats (N = 12). Representative photomicrographs show β-Tubulin III expression (red), ADNP (green), DAPI (blue), MERGE (yellow). Photomicrographs are representative results of fields taken randomly from each slide. Scale bar, 20 µm and 5 µm. The white arrows indicate spots showing exclusively ADNP immunoreactivity.

To investigate the cellular co-localization of ADNP and β-Tubulin III, we performed IF staining. As shown in Figure 5, in the DG of sedentary rats, ADNP and β-Tubulin III were mainly expressed in the perinuclear zone and in the cytoplasm. Moreover, they were not co-expressed by all cells, as clearly visible in the cells indicated by the white arrows. On the contrary, DG cells of rats subjected to moderate PA not only presented an increased expression of ADNP and β-Tubulin III, but these proteins were also always co-expressed in the perinuclear and cytoplasmic area of the analyzed cells.

**Figure 6:**
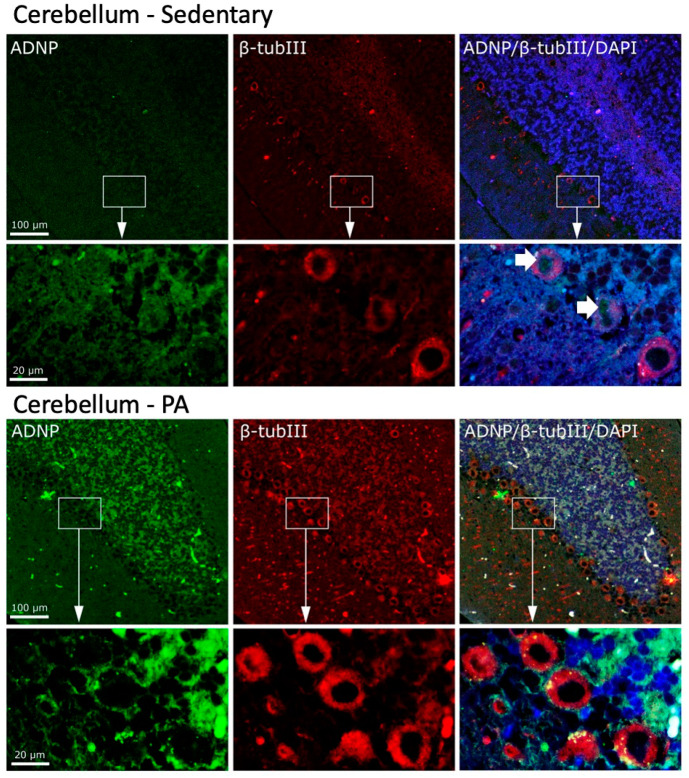
Co-localization of β-Tubulin III and ADNP in cerebellum of sedentary (N = 12) and active rats (N = 12). Representative photomicrographs show β-Tubulin III expression (red), ADNP (green), DAPI (blue), MERGE (yellow). Photomicrographs are representative results of fields taken randomly from each slide. Scale bar, 100 µm and 20 µm. The white arrows indicate spots showing nuclear ADNP immunoreactivity.

We then investigated the effect of moderate PA on the co-localization of ADNP and β-Tubulin III in the cerebellum. As shown in Figure 6, moderate and widespread immunoreactivity was detected of both ADNP and β-Tubulin III in the three cerebellar layers of sedentary rats. Interestingly, ADNP expression was found in the nucleus of Purkinje cells (indicated by white arrows), whereas, β-Tubulin III expression was detected in the perinuclear zone and in the cytoplasm of Purkinje cells. Active rats showed an increased expression and co-localization of ADNP and β-Tubulin III proteins in all cerebellar cortex layers. Furthermore, moderate exercise induced a marked change in the intra-cellular localization of ADNP protein, particularly evident in Purkinje cells: from predominantly nuclear localization in sedentary rats to predominantly cytoplasmic localization in active rats.

## 3. Discussion

Moderate aerobic exercise contributes greatly to improved cognitive function and overall brain health. PA enhances adult neurogenesis and synaptic plasticity, particularly in regions associated with learning and memory, such as the hippocampus, and motor learning and coordination, such as the cerebellum [34]. The positive effects of moderate PA on the brain are related to the modulation of different cellular and molecular mechanisms in the central and peripheral nervous systems. Each molecule or group of molecules released in response to exercise probably affects different aspects of brain function, but their synergistic effects contribute to overall brain health [17].

In the present study, we confirmed the positive role played by moderate aerobic exercise in terms of increased neuronal activation measured by the microtubule protein β-Tubulin III. This neuron-specific tubulin is involved in neurogenesis and is required for proper axon guidance [35,36,37]. Our data highlighted higher expression of β-Tubulin III in the DG area of the hippocampus of active rats as compared to sedentary animals. It is well known that during adult life, the DG is able to generate new neurons, which by becoming functionally active contribute to learning and memory [38,39]. The phenomenon of adult neurogenesis depends on different factors and stimuli. Among them, exercise is now considered one of the most effective inducers of adult neurogenesis in association with enhanced synaptic plasticity and memory function [6,40]. Beyond the DG area, adult neurogenesis occurs in other brain regions, including the cerebellum. In particular, evidence of adult cerebellar neurogenesis was demonstrated in transgenic mice exposed to physical activity [41,42]. Our results clearly show how rats performing moderate PA express higher levels of tubulin III in all layers of the cerebellar cortex, including increased branching of Purkinje cell dendritic trees, confirming the direct role played by aerobic exercise on the cerebellum.

Here, the novel aspect of this study was to investigate whether moderate PA modulates ADNP expression in specific areas of the rat brain. It is well known that ADNP is enriched in the brain, particularly in the hippocampus and cerebellum, compared to peripheral tissues. ADNP plays an important role in brain functioning, and it is necessary for proper brain development and to ensure its normal function [22]. It modulates several key genes linked to synaptic transmissions, such as ion channels, tubulin and microtubules, and genes regulating the autophagic process [43,44,45]. When one of the two copies of the *ADNP* gene is mutated and loses its normal function, the ADNP syndrome occurs, characterized by intellectual disability and global developmental delay which includes both language and motor domains [46]. In the present paper, we demonstrated for the first time that moderate PA significantly increased the expression of ADNP in the DG area of the hippocampus as compared to sedentary rats, whose DG cells did not always show strong immunopositivity staining for ADNP. This finding thus confirms the positive effects of PA. In fact, the increased hippocampal expression of ADNP was associated with better behavioral performance in intact male mice in terms of the social memory task. In contrast, *ADNP* haploinsufficiency provoked important cognitive impairment and delayed motor development [23]. Moreover, given that ADNP is dominantly localized near the subgranular zone, in future studies, it could be interesting to evaluate its co-localization with doublecortin (DCX) to investigate its involvement in adult neurogenesis.

Notably, the cerebellum is the main actor in coordinating motor movements including balance and motor learning. Here, we detected higher expression levels of ADNP in the cerebellum of active rats. Moreover, a most intriguing finding of the current study is the increased cytoplasmic distribution of ADNP in Purkinje cells of rats performing moderate aerobic exercise. It is well known that ADNP can be localized both in the nuclei and in the cytoplasm of neuronal and glial cells, since it contains both a nuclear localization signal and a leucine-rich nuclear export signal [19,47]. Moreover, previous studies showed the ability of ADNP to shuttle from the nucleus to the cytoplasm upon neuronal differentiation [48,49]. Interestingly, ADNP and the marker for developing neurons, β-Tubulin III, were co-expressed in both DG and cerebellar neurons of active rats compared to sedentary animals. These results suggest that moderate PA induces the interaction between ADNP and the brain-specific β-Tubulin III. A previous study demonstrated that the neuroprotection exerted by NAP, the active fragment of ADNP, is due to the specific interaction with β-Tubulin III essential for neuronal survival and function [22]. Therefore, we hypothesized that moderate PA increases ADNP expression and promotes its cytoplasmic localization, where interacting with β-Tubulin III induces neuroprotection.

One limitation of the present study was the use of the specific population of 3-month-old male Wistar Outbred Rats. Therefore, future studies will also expand to other developmental ages (e.g., peripuberty or late adolescence) and female rats. The latter aspect is very important considering that *ADNP* sex-dependently regulates multiple genes [50]. Very recently, Kapitansky and Gozes [51] investigated the effect of high-intensity resistance training on ADNP expression levels. They demonstrated that ADNP levels were significantly downregulated in vastus lateralis after prolonged endurance exercise, suggesting that after exercise, there is an increase in muscle size and strength, and the necessity for ADNP protective protein is reduced. In conclusion, the current work corroborates previous evidence that moderate aerobic exercise exerts a protective role, assuming that it could be partly mediated by up-regulation and interaction in specific brain areas between ADNP, necessary for proper brain functions, and β-Tubulin III, whose expression is related to neurogenesis, axon guidance, and maintenance.

## 4. Materials and Methods

### 4.1. Breeding and Housing of Animals

Twenty-four healthy 3-month-old male Wistar Outbred Rats were purchased from the Charles River Laboratories (Milan, Italy). Rats with an average body weight of 300 ± 20 g were housed in polycarbonate cages (10.25″ W × 18.75″ D × 8″ H) at 20–23 °C and controlled humidity. The rats were housed in a 12 h light/12 h dark cycle and received commercial feed and water ad libitum. On the day following the final training session (spanning a duration of 12 weeks), the rats were euthanized through intravenous administration of a lethal anesthetic overdose, utilizing a mixture of Zoletil 100 (Virbac, Milan, Italy) at a dosage of 80 mg/kg and DEXDOMITOR (Virbac, Milan, Italy) at a dosage of 50 mg/kg. Subsequently, the brains were extracted post-sacrifice, fixed in 4% paraformaldehyde for a minimum of 24 h, and then embedded in paraffin. All procedures adhered to the guidelines outlined by the Institutional Animal Care and Use Committee (I.A.C.U.C.) of the University of Catania (Protocol No. 2112015-PR dated 14 January 2015, approved by the Italian Ministry of Health). The experiments were conducted in compliance with the European Community Council Directive (86/609/EEC) and the Italian Animal Protection Law (Law No. 116/1992). The entire experimentation was performed at the “Center for Advanced Preclinical In Vivo Research (CAPIR)”.

### 4.2. Experimental Design

Twenty-four healthy 3-month-old male Wistar Outbred Rats were randomly divided into two groups, with 12 rats per group: sedentary rats (Group 1, sedentary) and rats undergoing moderate exercise on the treadmill (Group 2, PA). The programming of moderate PA follows the moderate physical training protocol for rats previously described by Di Rosa et al. [52]. In particular, Group 2 performed 12-week running training. The rats ran 5 days a week for 20 min daily on a treadmill (2Biological instrument, Varese, Italy) with 2° slope set to an incremental speed that ranged 10–30 m/min to perform a moderate-intensity exercise. On the day following the last training (after 12 weeks of the experiment), the animals were humanely sacrificed by a lethal intravenous injection of anesthetic overdose, and brain samples were explanted and fixed for the immunohistochemical analysis and Western blot analysis.

### 4.3. Immunohistochemistry (IHC) Analysis

The expression and distribution of ADNP and β-Tubulin III in the DG and cerebellum of sedentary and PA rats was evaluated through immunohistochemical analysis as previously described [53]. The sections were incubated overnight at 4 °C with the specific antibodies: rabbit anti-ADNP (NBP1-89236); mouse anti- β-Tubulin III (ab78078). The immunoreaction was visualized using a 3,3′-diaminobenzidine solution (DAB substrate Kit; SK-4100, Vector Laboratories, Burlingame, CA, USA). Following this, the samples underwent a light counterstaining with hematoxylin. Observation was carried out utilizing an Axioplan Zeiss light microscope (Carl Zeiss), and the digital micrographs were captured using a digital camera (AxioCam MRc5, Carl Zeiss) via AxioVision Release 4.8.2—SP2 Software (Carl Zeiss Microscopy GmbH, Jena, Germany). Original microphotographs are reported in Figure S1 (scale bar 50 μm and 10 μm).

The percentage of area stained with β-Tubulin III or ADNP antibody was calculated by using ImageJ software (NIH, Bethesda, MD; available at http://rsb.info.nih.gov/ij/index.html, accessed on 9 January 2024), which quantifies the level of staining intensity of positive immunolabeling in each field. The statistical analysis details are reported in Table S1.

### 4.4. FFPE Tissue Samples

Formalin-fixed, paraffin-embedded 16 μm thick tissue sections from the hippocampus and cerebellum of group 1 (sedentary) and group 2 (PA) were collected in tubes. The extraction of proteins was performed through Qproteome FFPE Tissue Extraction Buffer (Qiagen, Hilden, Germany). Briefly, tissue sections were deparaffinized with xylene and rehydrated with a graded ethanol series (100%, 96%, and 70%). An extraction buffer, containing β-mercaptoethanol, was added to the pellet of each tube and incubated at 100 °C for 20 min and at 80 °C for 2 h. Then, the tubes were centrifuged for 15 min at 14,000× *g*. The supernatants containing the extracted proteins were collected and stored at 4 °C. Protein concentrations were evaluated by using the Quant-iT Protein Assay Kit (Invitrogen, Carlsbad, CA, USA).

### 4.5. Western Blot Analysis

About 15 μg of proteins from formalin-fixed tissue sections were diluted in 2× Laemmli buffer (Invitrogen, Carlsbad, CA, USA), heated at 70 °C for 10 min, loaded on 4–12% tris–glycine gel, and then transferred to a nitrocellulose membrane as previously described [54]. Blots were blocked for 1 h using the Odyssey Blocking Buffer (Li-Cor Biosciences). Then, the membranes were incubated overnight (at 4 °C) with specific primary antibodies: rabbit anti-ADNP (NBP1-89236); mouse anti- β-Tubulin III (ab78078); mouse anti- β-actin (sc-47778). The secondary antibody, goat anti-rabbit IRDye 800CW (926-32211; Li-Cor Biosciences) and goat anti-mouse IRDye 680CW (926-68020D; Li-Cor Biosciences), was used at 1:20,000. Blots were scanned with an Odyssey Infrared Imaging System (Odyssey, Li-Cor Biosciences, Lincoln, NE, USA). Original images are reported in Figure S2. ImageJ software was used for the densitometric analyses of Western blot signals. Values were normalized to β-actin used as a loading control. The statistical analysis details are reported in Table S1.

### 4.6. Immunofluorescence Analysis

To evaluate the cellular distribution of co-localization of ADNP and β-Tubulin III in the DG and cerebellum of sedentary and PA rats, immunofluorescence analysis was performed as previously described by Maugeri et al. [55]. The sections were incubated overnight at 4 °C with the specific antibodies: rabbit anti-ADNP (NBP1-89236) and mouse anti- β-Tubulin III (ab78078). Signals were revealed with Alexa Fluor 488 goat anti-mouse and Alexa Fluor 594 goat anti-rabbit, for 1.5 h at room temperature (shielded from light). DNA was counter-stained with 4,6-diamidino-2-phenylindole (DAPI; cat. no 940110; Vector Laboratories, Burlingame, CA, USA). Immunolocalization was analyzed by confocal laser scanning microscopy (Zeiss LSM700, Oberkochen, Germany). Original microphotographs are reported in Figure S3.

### 4.7. Statistical Analysis

Data were analyzed using GraphPad Prism 9 (GraphPad Software, La Jolla, CA, USA). All values are presented as means ± SEM. Distribution normality was assessed using the Shapiro–Wilk test. Statistical significance was assessed via an unpaired two-tailed Student’s *t* test. The level of significance for all statistical tests was set at *p* ≤ 0.05. Details concerning statistical test are reported in the Supplementary Materials Table S1.

## Data Availability

Data is contained within the article and Supplementary Materials.

## Supplementary Materials

The following supporting information can be downloaded at: https://www.mdpi.com/article/10.3390/ijms25084382/s1.
