# Supplementary material for: Moderate Physical Activity Increases the Expression of ADNP in Rat Brain
Source: Int J Mol Sci. 2024 Apr 16;25(8):4382. doi: 10.3390/ijms25084382 (PMC11050439; doi:10.3390/ijms25084382)
Supplement: Supplementary file 1 [file ijms-25-04382-s001.zip › ijms-2940493-supplementary.pdf]

SUPPLEMENTARY DATA

Table S1. Statistical Analysis Details.

Fig. #	Statistical test	Factors, Degree of freedom & F/t value	Significance
1B	Unpaired t test (Two-tailed)	t=5.082, df=22	****
2B	Unpaired t test (Two-tailed)	t=11.84, df=22	****
3C	Unpaired t test (Two-tailed)	t=10.69, df=22	****
4D	Unpaired t test (Two-tailed)	t=14.48, df=22	****
1C	Unpaired t test (Two-tailed)	t=10.57, df=22	****
2C	Unpaired t test (Two-tailed)	t=7.616, df=22	****
3C	Unpaired t test (Two-tailed)	t=13.46, df=22	****
4D	Unpaired t test (Two-tailed)	t=11.90, df=22	****

****$p<0.0001$ vs. sedentary, as determined by unpaired two-tailed Student t-test.

Figure S1. IHC original microphotographs.

Scale Bar: 50 μm and 10 μm.

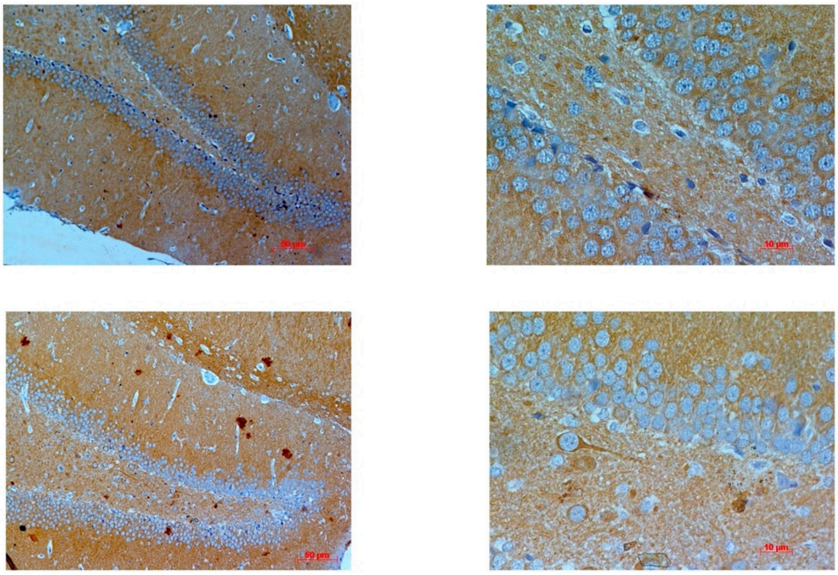

Figure 1

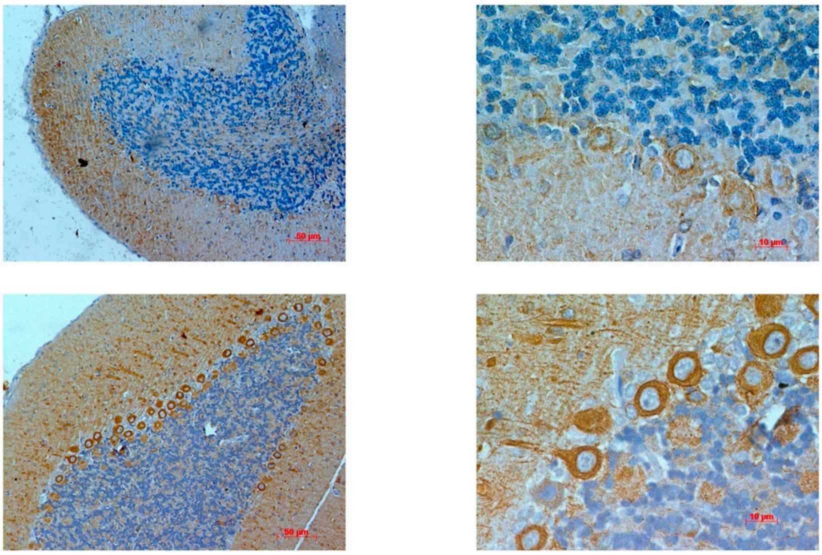

Figure 2

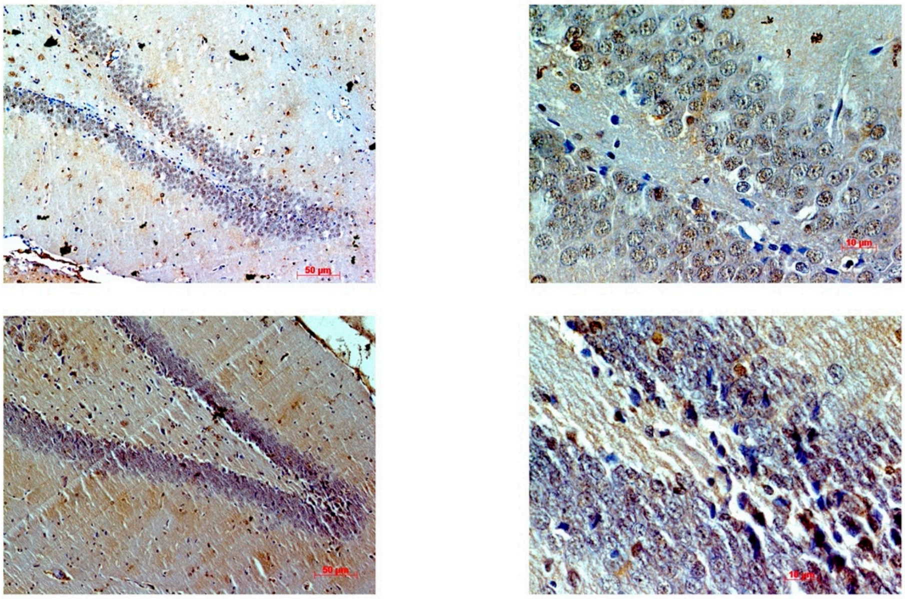

Figure 3

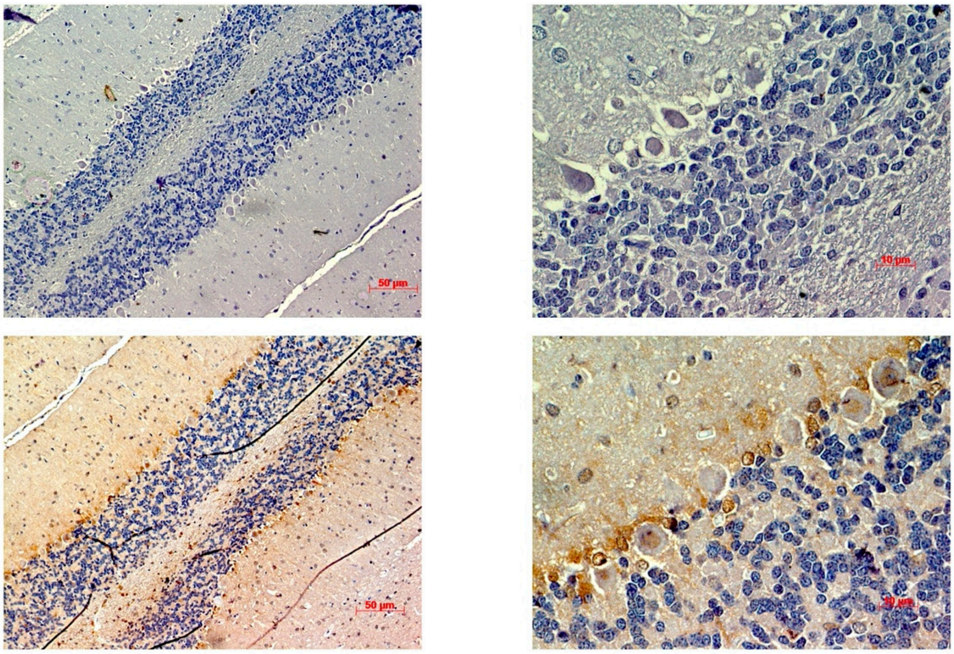

Figure 4

Figure S2. Original western blot.

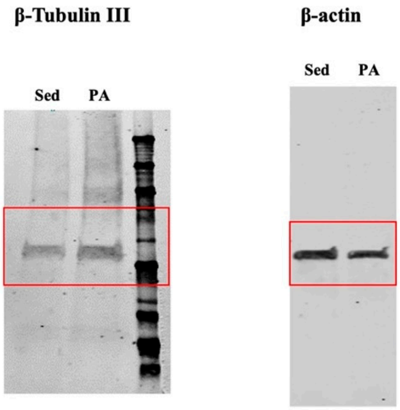

Figure 1

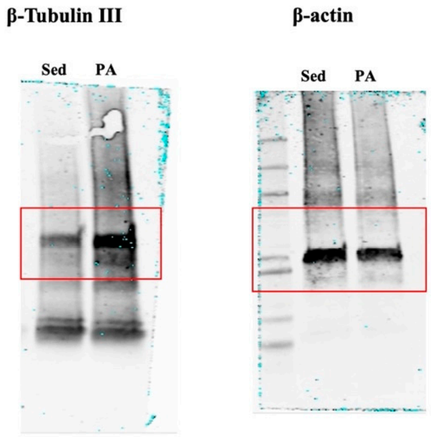

Figure 2

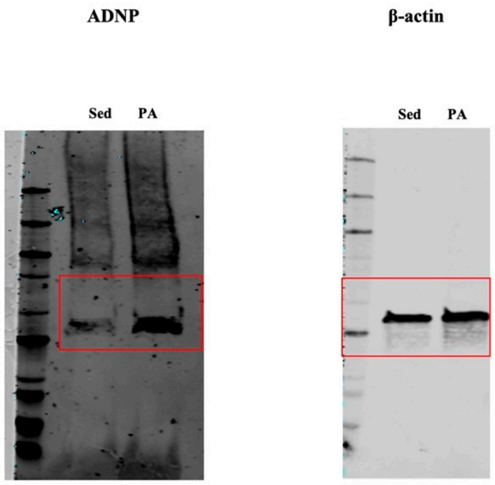

Figure 3

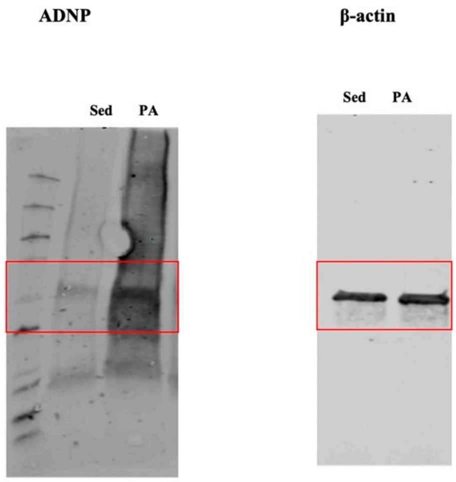

Figure 4

Figure S3. IF original microphotographs.

DG sedentary (scale bar: 20 μm and 5 μm)

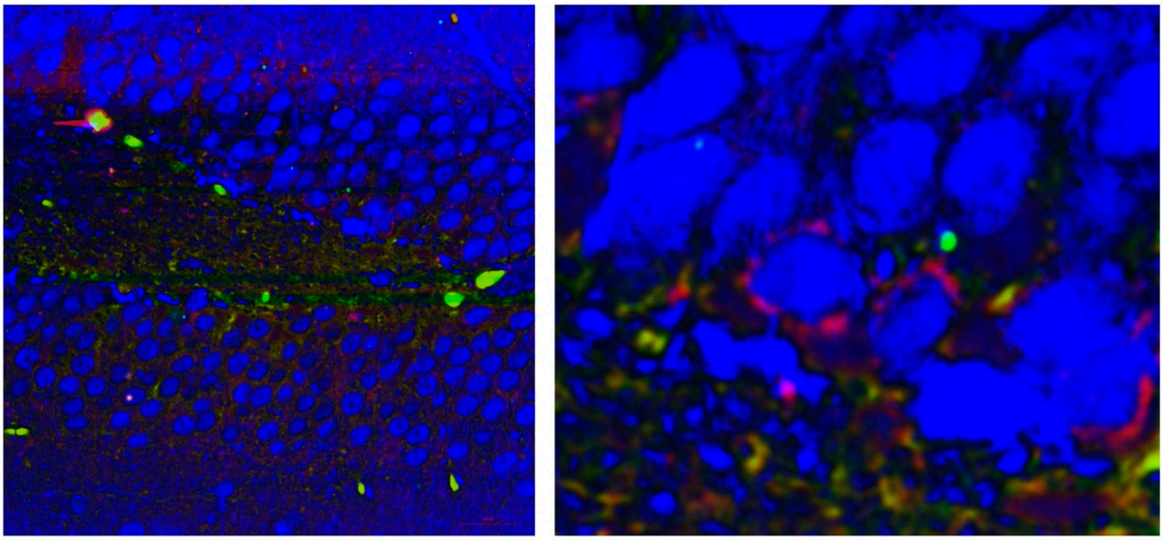

Figure 5 – DG sedentary

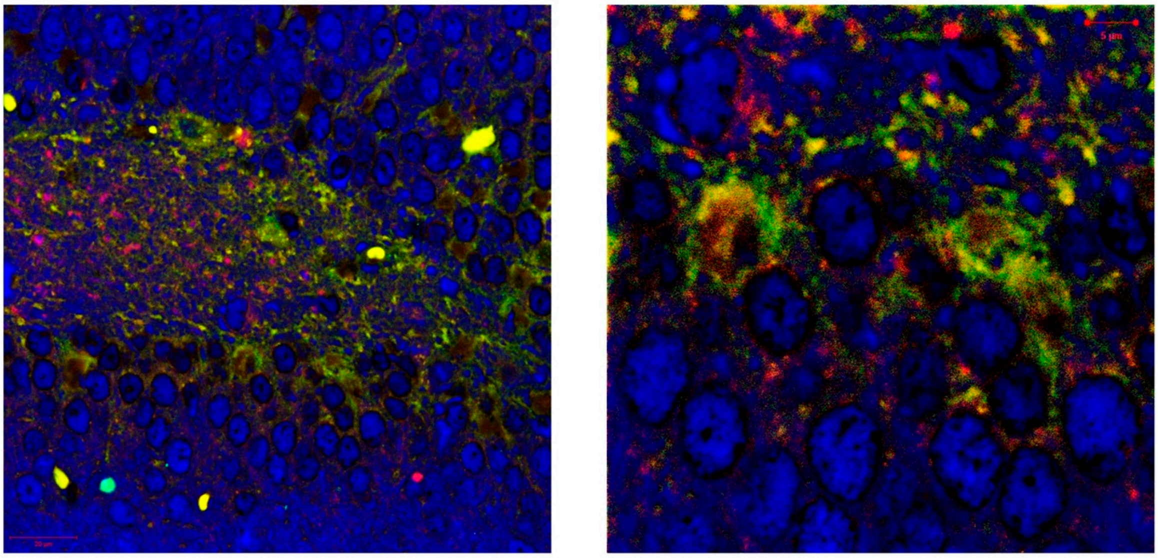

Figure 5 – DG PA

DG PA (scale bar: 20 µm and 5 µm)

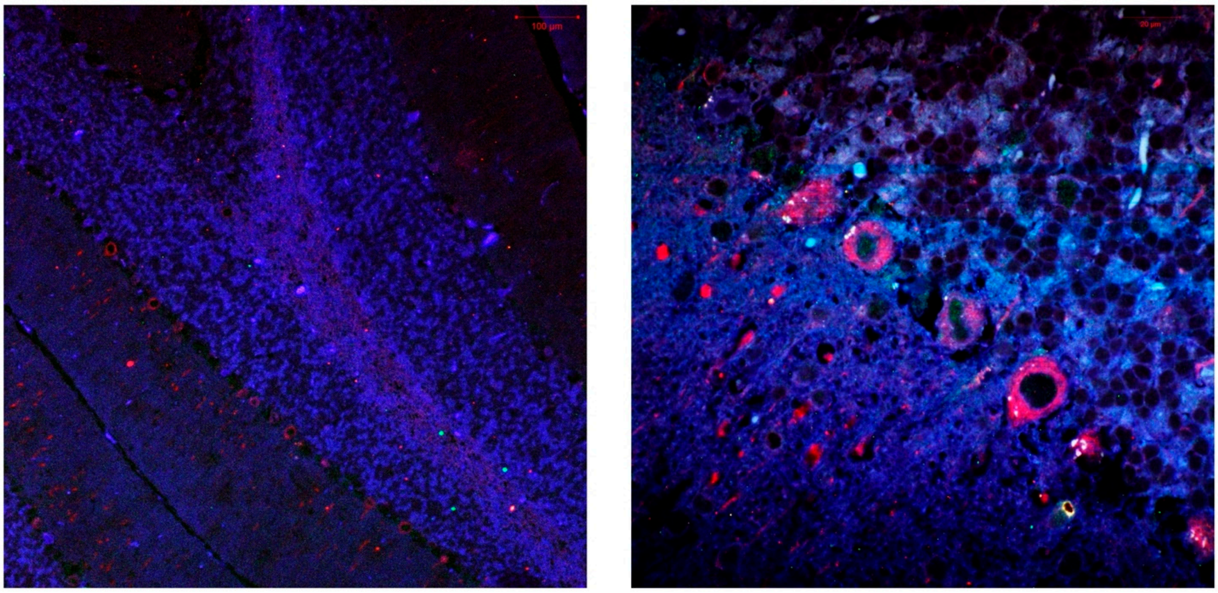

Figure 6 – cerebellum sedentary

cerebellum sedentary (scale bar: 100 µm and 20 µm)

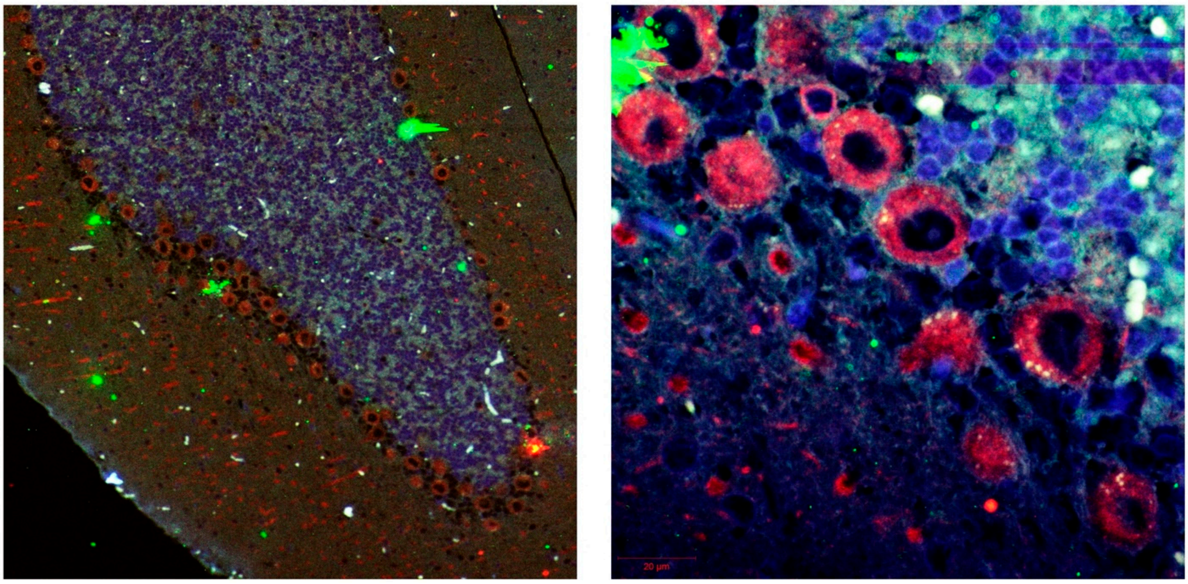

Figure 6 – cerebellum PA

Cerebellum PA (scale bar: 100 µm and 20 µm).
